# Effect of Time of Temperature Exposure on Routine Metabolic Rate and Body Mass Scaling in Alpine Charr (*Salvelinus umbla*)

**DOI:** 10.1002/ece3.71907

**Published:** 2025-08-06

**Authors:** Allan Raffard, Martin Daufresne, Jean Guillard, François‐Raphaël Lubin, Emilie Réalis‐Doyelle, Hervé Rogissart, Loïc Teulier

**Affiliations:** ^1^ Univ. Savoie Mont Blanc INRAE, CARRTEL Thonon‐les‐Bains France; ^2^ Pôle ECLA (OFB, INRAE, USMB) Thonon‐les‐Bains France; ^3^ INRAE, Aix‐Marseille Univ, RECOVER Aix‐en‐Provence France; ^4^ Universite Claude Bernard Lyon 1 CNRS, ENTPE, LEHNA UMR 5023 Villeurbanne France

## Abstract

The thermal dependence of metabolism has attracted much attention because metabolism is linked to many ecological properties, from individuals to ecosystems. The rate at which physiological traits change is an important factor to account for when assessing thermal plasticity since the time of exposure might allow organisms to compensate for the effect of temperature on performance. Here, we studied short‐ and long‐term thermal plasticity of metabolic rate and its scaling with body mass of Alpine charr ( *Salvelinus umbla* ). To do so, we raised juveniles from Lake Geneva at 4.5°C and 8.5°C, measured their routine metabolic rate at their incubation temperature (long exposure, > 6 months), and individuals raised at 4.5°C were also tested at 8.5°C (short exposure, < 24 h). The metabolic rate of Alpine charr increased with temperature, as theoretically expected. We found no effects of duration of exposure on metabolic rate or its scaling with body mass. Despite a long‐term exposure to higher temperature, individuals did not adjust their metabolic rate as compared to individuals exposed to a rapid increase in temperature, questioning the capacity of Alpine charr to compensate for temperature increase through their metabolic rate. Therefore, our data reveal no compensation mechanisms of Alpine charr to counterbalance the acute effect of temperature. Further studies will be required to fully understand the adaptive potential of Alpine charr in the context of global change.

## Graphical abstract

The rate at which phenotypic plasticity occurs is now acknowledged to be an important factor to account for when studying plasticity, as it allows to understand how organisms can respond to rapid environmental changes. Using Alpine charr (*Salvelinus umbla*) as a model species, we found that time of exposure to higher temperature did not alter metabolic rate or its scaling with body mass. Interestingly, Alpine charrs are considered stenothermic species, and our results suggest that their adaptive capacities and their potential evolution in warmer and less predictable environments might be questioned.

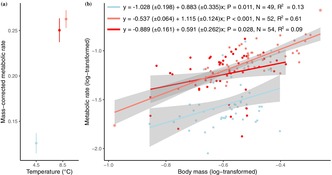

## 1 Introduction

Defined as the capacity of a genotype to produce different phenotypes, phenotypic plasticity permits rapid change in morphological, behavioral, and physiological traits, allowing organisms to cope with environmental variations, and can alter interactions in communities (DeWitt and Scheiner 2004; Stearns 1989; Turcotte and Levine 2016). Plasticity is a ubiquitous process exhibited by many kinds of morphological, behavioral, and physiological traits (Dingemanse et al. 2010; Jacob and Legrand 2021; Kar et al. 2022). Among other traits, many studies have focused on the variability of metabolic rate. Because it is closely related to energetic requirements, this trait has been suggested to drive many ecological properties, from individuals to ecosystems (Brown et al. 2004; Burton et al. 2011; Raffard et al. 2023; Schaum et al. 2018; Seebacher et al. 2015). In addition, metabolic rate has long been known to increase with temperature, following an Arrhenius law (Arrhenius 1915). From this viewpoint, the study of metabolic plasticity has improved the understanding of the energetic requirements of individuals and species persistence in response to changes in temperature, which is important in the current context of global change (Seebacher et al. 2015).

Importantly, the rate of phenotypic change (including metabolic rate) is a neglected dimension of phenotypic plasticity (Burton et al. 2022; Dupont et al. 2024). The temporal dynamic of a change in a trait value can indeed vary (e.g., across individuals, taxa or traits), and the change over time can follow different shapes (e.g., linear or nonlinear temporal dynamics; Burton et al. 2022; Dupont et al. 2024). In ectothermic organisms, the rate of phenotypic change is particularly dependent upon the temperature and can vary among taxa. For instance, among ectotherms, the rate of thermal tolerance changes in response to changes in temperature is lower in fishes, meaning that phenotypic changes take more time in fishes than in amphibians, reptiles, or other ectothermic individuals (Einum and Burton 2022). Briefly, this rate is dependent upon the detection of information and the subsequent biochemical mechanisms involved in the response, including gene expression and production of proteins, for example, that involve an incompressible amount of time (Burton et al. 2022; Dupont et al. 2024). As a consequence, many reaction norms are determined by the time of exposure to an environment. Rapid changes in temperature can induce “passive” plasticity due to the thermal dependence of chemical reactions (Schulte et al. 2011). However, prolonged acclimation periods allow organisms to exhibit active plasticity (including developmental plasticity), which might decrease the thermal effects on the trait (Havird et al. 2020; Schulte et al. 2011). Compensation has been described as the process leading to the return of a physiological rate to its original value, at least partially (Havird et al. 2020). Acclimation period and the potential of species to adjust their phenotype to novel environmental conditions may be critical in nature since thermal conditions are expected to be less predictable and more variable in the future due to climate change (Easterling et al. 2000).

While metabolic rate varies according to temperature, it is also strongly linked to individual body mass (Gillooly 2001). The metabolic theory of ecology indeed historically predicts a universal scaling exponent between resting metabolic rate and body mass, mainly due to physical and chemical constraints (Brown et al. 2004). Nonetheless, several studies are now reporting that these scaling laws may actually be variable (Glazier 2005, 2022). Among other factors, the scaling exponent can vary because temperature or lifestyle of organisms, such as predation rate or activity, which can modify the mass‐specific energetic requirement of large and small individuals, subsequently altering the scaling exponent (Killen et al. 2010). Such interactions between body mass and temperature on metabolic rate are common and have been observed both among and within species (Glazier and Gjoni 2024; Killen et al. 2010). Interesting studies have shown, for example, that fish predation can decrease the scaling between body mass and metabolic rate (Glazier et al. 2011), but that this effect can be mediated by temperature regimes (Glazier et al. 2020). Temperature is indeed central in mediating the scaling of metabolic rate with body mass (Glazier and Gjoni 2024). Since it is expected that the rate of change in metabolic rate is dependent on acclimation time and would likely change faster than body mass, it is likely that acclimation time to a new temperature regime might alter the linkage between mass and metabolism. Studying metabolic plasticity, the effect of acclimation to warm temperature, and the linkage with body mass is important to better understand future responses of species to climate change (Brown et al. 2004; Seebacher et al. 2015).

In this study, we investigated rapid and long‐term thermal plasticity of metabolic rate and its relationship with body mass. Specifically, juvenile Alpine charrs (*Salvelinus umbla*), a cold‐water fish, were incubated at 4.5°C and 8.5°C, and we quantified routine metabolic rate (RMR) of fish acclimated to these temperatures. Additionally, rapid phenotypic change was assessed by measuring RMR at 8.5°C in fish incubated at 4.5°C. Rapid changes in temperature are indeed predicted to increase with climate change (Easterling et al. 2000). In this context, heat shocks are particularly stressful for stenothermic populations (Massey and Hutchings 2021), with long‐term impact on individual phenotype (Lubin et al. 2024). Here, we specifically expected a higher RMR at warmer temperatures (Gillooly 2001; Huuskonen et al. 2003), and that rapid increase in temperature should lead to a higher RMR than long‐term increase because adaptive physiological responses allowing for compensation of the temperature effect may involve a longer time lag (Moffett et al. 2018). Moreover, since the scaling of metabolism with body mass is expected to vary in response to temperature, as predicted by the metabolic‐level boundaries hypothesis (Glazier 2014, 2005; Killen et al. 2010), it should differ among fish from the different treatments.

## 2 Material and Methods

### 2.1 Ethics Statement

This project was approved by an ethic committee (#015) in accordance with Directive EU 2010/63/EU (authorization #40219‐2023012316461089 v1).

### 2.2 Study Species

Arctic charr (*Salvelinus* sp.) forms a species complex distributed in circumpolar areas, including Asia, North America, and north of Europe (Klemetsen 2010, 2013). In the Arctic and pre‐Arctic region, it is often referred to as Arctic charr ( *S. alpinus* ), while in the Alps, it can be referred to as Alpine charr ( *S. umbla* ) (Keith et al. 2011; Kottelat and Freyhof 2007). Here, we focus on Alpine charr from Lake Geneva, which have colonized deep areas to meet optimal temperatures for reproduction (Dussart 1955). Since reproduction takes place during the winter, we collected reproductive individuals from professional fishers in December 2022 in order to gather gametes. In Lake Geneva, supportive breeding was the primary method of stocking from mid‐November to the end of December (Caudron et al. 2014). Wild spawners were captured using nets in the primary spawning locations in France by professional fishers (Caudron et al. 2014). Five full‐sib families (hereafter referred to as L1, L3, L4, L8, and L10) were obtained by mixing milt and eggs from five males and five females (body length of reproductive individuals ranged from 32 cm to 46 cm, Table S1), respectively. An average of 82 (±11 standard deviation [SD]) eggs per family were incubated at 4.5°C, and 90 (±9 SD) eggs at 8.5°C (see Table S2 for the sample size per families). These temperatures correspond to an optimal temperature range for the incubation period without significantly increasing mortality (Guillard et al. 1992). Eggs from different families were incubated separately in 5 × 5 × 5 cm boxes placed in 20 L tanks until hatching. Water was aerated to 100% O_2_ saturation using an air pump, and temperature was controlled using an aquarium chiller (TK1000; TECO, Ravenna, Italy). At 4.5°C, fish hatched at 414 (±7.6 SD) accumulated degree days (ADD) with a mean hatching rate of 86.3% (±6.7 SD) (Table S2). At 8.5°C, fish hatched at 441 (±9.8 SD) ADD with a mean hatching rate of 75.8% (±11 SD). Ten individuals per family were pictured (using a Nikon D5300), and pictures were analyzed with ImageJ (Schneider et al. 2012) to obtain body size at hatching.

After hatching, fish from each family were separately placed in 2.5 L tanks (i.e., one tank per family per temperature of incubation so as to control for potential biases induced by stochasticity of other factors such as environmental condition; Réalis‐Doyelle et al. 2016), kept at 4.5°C or 8.5°C in two thermostatic chambers (Réalis‐Doyelle et al. 2023). Each family was raised at both temperatures so as to allow comparison of metabolic rate among acclimation and thermal conditions. Water in tanks was recirculating using a pump (Eiheim universal 3400), continuously aerated to ensure optimal oxygen conditions, treated with a UV lamp (AQUA NOVA Sterilizer), and filtered (JBL blue filter foam). Two‐thirds of the water volume was changed every day to limit nutrient accumulation in the system, and we monitored physico‐chemical characteristics of the water twice a week. Fish were kept in a light–dark cycle of 12:12 h.

### 2.3 Routine Metabolic Rate Measurement

From July 2023, routine metabolic rates were individually assessed using eight acrylic cylindrical chambers (Loligo Systems, Viborg, Denmark) (∅ 42 mm × 52 mm, 70 mL, 120 mL with pipes) immersed in a fully aerated water tank, regulated at 4.5°C and 8.5°C using a TECO water chiller (TK 1000; TECO, Ravenna, Italy). Oxygen concentration in each chamber was measured every second using optodes connected to an oxygen meter (Witrox‐4; Loligo Systems, Viborg, Denmark) with a specific software (WitroxView; Loligo Systems, Viborg, Denmark V2.0.3).

Fish incubated at 8.5°C were tested at 8.5°C (warm acclimation—warm test), and fish incubated at 4.5°C were separated into two distinct groups: one group was tested at 4.5°C (cold acclimation—cold test) and the other at 8.5°C (cold acclimation—warm test) to test the effect of a rapid temperature increase on metabolism. To do so, fish from the cold acclimation—warm test group were transferred from 4.5°C water to 8.5°C water within a few minutes; rapid changes in temperature did not affect mortality and are common in nature (Lubin et al. 2024; Rautureau et al. 2025). For logistic reasons, RMR was estimated once for each condition, with metabolic measurements carried out between 1313 and 1542 ADD and between 1964 and 2801 ADD for fish incubated at 4.5°C (cold acclimation—cold test and cold acclimation—warm test) and 8.5°C, respectively. This difference is unlikely to alter the conclusions of this study since all fish were at the same developmental stage (juvenile), and the correlation between ADD and metabolic rate within each condition was not significant.

Before placing the fish in the chambers, and given the relatively young stage of individuals, they were fasted for 24 h to ensure a similar nutritional state of all individuals (Alberto‐Payet et al. 2022; Chabot et al. 2016; Eliason and Farrell 2016; Secor 2009). Following an intermittent‐stop flow protocol (Killen et al. 2021), fish were placed every day at the same time (approximately 11.00 am) in the chambers for approximately 20 h. Every 90 min, water from the chambers was flushed using an automated pump (New Jet NJ1700; Newa, Italy) during 3 min to increase oxygen concentration in the chambers to 100% saturation. The durations of flush and measurement periods were characterized in previous preliminary experiments so that we ensured that the oxygen concentration did not reach stressful concentrations (the saturation of oxygen was never below 80%). The metabolic trials were conducted in the dark to reduce stress for the fish. After the test, all individuals were weighed to the nearest 0.001 g. The min‐max range of body mass was 0.105–0.548 g.

The decrease in oxygen concentration in a 50‐min interval within the 90‐min closed period was used to estimate the metabolic rate after an initial 25‐min waiting period. Background bacterial oxygen consumption was assessed during 1 h before and after each test in all chambers. Bacterial correction of metabolic rate (mgO_2_·h^−1^) was calculated using the function *adjust_rate* of the *respR* R‐package (Harianto et al. 2019). Because the slopes between O_2_ concentration and time were rather low, the proportion of variance due to noise variability might decrease the *R* ^2^ (Svendsen, Bushnell, Christensen, and Steffensen 2016). Therefore, for each individual, all slopes (between oxygen concentration and time) with an *R* ^2^ > 0.85 were averaged to get the mean consumption of oxygen for each individual (Chabot et al. 2021; Svendsen, Bushnell, Christensen, and Steffensen 2016; Svendsen, Bushnell, and Steffensen 2016). To control for a potential stress of handling, we also removed the first slope, corresponding to the first enclosed period of 90 min, where consumption of oxygen was quantified.

### 2.4 Statistical Analyses

First, we tested whether size at hatching differed between fish incubated at 4.5°C and 8.5°C using a mixed‐effect linear model (LMME, *lmer* function from the *lme4* R‐package; Bates et al. 2014). Size at hatching was set as the response variable, and incubation temperature as the explanatory variable, and the family was set as a random effect. Second, we used LMME to test whether acclimation treatment affects metabolic rate and its scaling with body mass. The log‐transformed RMR (mgO_2_·h^−1^) using base 10 logarithms was set as the response variable, the acclimation treatment (3‐level factors representing warm acclimation—warm test; cold acclimation—cold test; cold acclimation—warm test), the log‐transformed body mass (g) and their interaction as explanatory variables, and the family was included as a random parameter. We assessed pairwise mean differences between treatments using post hoc contrasts (using the *emmeans* function from the R‐package *emmeans*; Length et al. 2022). Finally, we calculated Q10 for long‐term and short‐term acclimation conditions as Q10 = (RMR_8.5_/RMR_4.5_) ^(10/8.5–4.5)^, where RMR_8.5_ and RMR_4.5_ were routine metabolic rates at 8.5°C and 4.5°C, respectively.

All statistical analyses were carried out using R (R Core Team 2013).

## 3 Results

**FIGURE 1:**
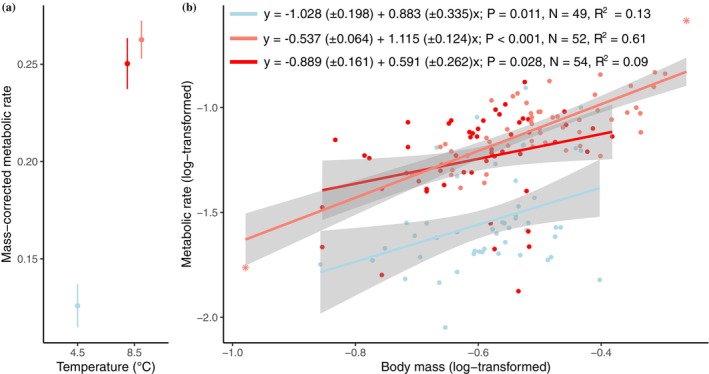
(a) Effect of temperature and acclimation temperature on metabolic rate (mgO_2_·h^−1^·g^−1^) of Alpine charr ( *Salvelinus umbla* ). Error bars represent ±1 standard error SE. (b) Relationship between body mass (g, log‐transformed) and metabolic rate (mgO_2_·h^−1^, log‐transformed) in each of the acclimation conditions. Stars represent extreme points. Shaded areas represent 95% confidence intervals CIs. Blue points, error bars, and slopes represent the cold acclimation—cold test condition; dark red points, error bars, and slopes represent cold acclimation—warm test condition; and the light red points, error bars, and slopes represent the warm acclimation—warm test conditions. The regression equations were calculated based on a linear model (numbers into brackets are SE).

Overall, fish were longer at hatching when incubated at 4.5°C compared to the fish incubated at 8.5°C (*χ* ^2^ = 522.84, df = 1, *p*‐value < 0.001). We found a significant thermal plasticity of metabolic rate, as highlighted by the significant contrasts between metabolic rate measured at 4.5°C and 8.5°C (Table 1). Nonetheless, the thermal plasticity of metabolic rate was not affected by the acclimation period (Figure 1a). Indeed, temperature significantly increased the metabolic rate of individuals, regardless of the acclimation period (Figure 1a). Short‐term and long‐term acclimation to high temperature induced Q10 of similar strength (5.57 and 6.28, respectively).

**TABLE 1:** Results of the pairwise contrasts comparing the routine metabolic rate of Alpine charr ( *Salvelinus umbla* ) juveniles among acclimation conditions and test temperature.

Pairwise contrasts	Difference estimate	SE	df	*t*	*p*
**Cold acclimation—cold test vs. Cold acclimation—warm test**	**−0.304**	**0.038**	**145**	**−8.152**	**< 0.001**
**Cold acclimation—cold test vs. Warm acclimation—warm test**	**−0.362**	**0.038**	**145**	**−9.414**	**< 0.001**
Cold acclimation—warm test vs. Warm acclimation—warm test	0.056	0.038	146	−1.482	0.302

*Note:* Fish were acclimated to either 4.5°C (Cold acclimation) or 8.5°C (Warm acclimation) and tested at the same or a different temperature (cold test, 4.5°C or warm test, 8.5°C). Significant effects are displayed in bold.

The slopes between body mass and metabolic rate were similar across the treatments of temperature and acclimation periods, as indicated by the nonsignificant interaction between the treatment of temperature and body mass (*χ* ^2^ = 1.521; df = 2; *p*‐value = 0.467, Table 2, Figure 1b). Finally, the random term related to the inter‐family variability in mean RMR explained 8% of the total variance (see also Figure S1).

**TABLE 2:** Effects of body mass, acclimation conditions, and their interaction on routine metabolic rate (mgO_2_·h^−1^, log‐transformed) of Alpine charr ( *Salvelinus umbla* ) using a linear model with mixed effect (with the family as a random factor).

Effect	*χ* ^2^	df	*p*
**Body mass**	**49.670**	**1**	**< 0.001**
**Acclimation condition**	**120.016**	**2**	**< 0.001**
Body mass * Acclimation condition	1.521	2	0.468

*Note:* Significant effects are displayed in bold.

## 4 Discussion

We found that the routine metabolic rate of Alpine charr was higher at warmer temperatures, denoting the thermal plasticity of this trait. Nonetheless, the metabolic rate was not dependent on the time of exposure to warm temperatures, nor was its relationship with body mass. Acclimation time may alter the quantification of phenotypic plasticity because phenotypic changes are not instantaneous and may take time to occur. Nonetheless, our findings did not confirm this assumption.

While we primarily hypothesized that a rapid increase in temperature would lead to a higher metabolic rate compared to long‐term exposure, our findings suggest that acclimation time did not alter the mean response of metabolic rate to warming. Particularly, the short‐term acclimation treatment provides a test for reversible plasticity, while the long‐acclimation condition (both cold and warm) also encompasses developmental plasticity since individuals were exposed to different temperatures since fertilization. Early‐life environment can indeed induce long‐term changes in an individual's physiology (Kar et al. 2022; O'Dea et al. 2019). Acclimation time and developmental environment may lead to compensation in physiological processes that involve active mechanisms (Havird et al. 2020). This compensation might allow individuals to maintain a physiological process at an optimal rate and is probably due to adaptive mechanisms (Fangue et al. 2009; Schulte et al. 2011). While the positive effect of temperature on metabolic rate was theoretically predicted, the nonsignificant difference between long‐term and short‐term acclimation suggests that developmental plasticity in some traits, such as metabolism, can be reversible, allowing for rapid change following environmental conditions. Some studies suggest that developmental temperature does not affect thermal plasticity of metabolism (Kar et al. 2022); our results tend to support this conclusion. Since physiological traits are mostly labile, performing reciprocal crosses from warm to cold temperatures might generate insightful findings regarding cold acclimation of metabolic rate.

We found similar Q10 in both conditions, which confirms that fish acclimated to higher temperature were not able to adjust their routine metabolic rate to higher temperature (Havird et al. 2020). One possibility is that compensation might occur only in stressful conditions (e.g., lack of resources) to optimize energetic expenditure. Therefore, under optimal feeding conditions, as in our study, a higher metabolic rate might not be a disadvantage. This first finding may also suggest that the thermal plasticity of routine metabolic rate in Alpine charr relies mostly on passive reactions (i.e., due to the thermodynamics of biochemical processes). It is important to note that our results are specific to routine metabolic rate. While RMR was probably dependent on temperature passively, other aspects of metabolism, such as maximal metabolic rate, aerobic scope, or standard metabolic rate, might involve active adaptive mechanisms to lower the effect of temperature on maximal energetic requirement. For instance, the mitochondrial oxygen consumption responds more rapidly to acute increases in temperature in warm‐adapted than in cold‐adapted killifish ( *Fundulus heteroclitus* ) (Fangue et al. 2009). Investigating other aspects of metabolism might hence bring interesting insights regarding the role of acclimation time and active plasticity in Alpine charr.

Our results also suggest that the scaling between body mass and metabolic rate may be conserved across temperature and acclimation time. We indeed found a classical positive slope between RMR and body mass. The conservation of the allometric coefficient has long been debated. It can indeed vary depending on many environmental factors (e.g., temperature), leading to plasticity, evolution, and/or acclimation (Glazier 2005). Nonetheless, the slope did not vary in our study, and multiple hypotheses are possible. First, while temperature is important to mediate metabolic rate and its scaling with body mass, many internal factors can mediate its effect (Glazier and Gjoni 2024). Temperature can indeed affect other traits concomitantly with metabolism, such as activity. While space was limited in the chamber, it is a hardly controllable parameter, and temperature may have induced an increase in activity rate. For example, Glazier (2020) has shown that activity (and growth rate) may prevent declines in the scaling exponent predicted by the metabolic‐level boundaries hypothesis for resting metabolic rate, especially in mobile species. It is also possible that the 90‐min recovery period after handling was not sufficient to allow full physiological recovery; therefore, our measure of RMR might include a small proportion of stress. Second, while we primarily chose a conservative and realistic temperature delta (4°C), it may be too small to detect an effect on the scaling exponent. Other studies have, for instance, considered a delta up to 12°C (Glazier et al. 2020).

Overall, large individual variation in metabolic rate observed in our data may have prevented observations of any significant effects of temperature on the metabolic scaling exponent. Intraspecific variation in metabolic rate results from several factors (Burton et al. 2011), such as bet hedging, developmental noise, and maternal effects (Burton et al. 2011; O'Dea et al. 2019). This variation is key in determining fitness and performance of individuals, especially to face environmental changes (Noble et al. 2025; Rota et al. 2025). Nonetheless, variance in metabolic rate, especially around the scaling estimates, can result in low power to detect significant differences. Especially, the standard deviation in mass‐corrected metabolic rate was slightly higher in the short‐term acclimation condition (SD = 0.096) as compared to cold and warm acclimated conditions (SD = 0.078 and 0.070, respectively). Although acclimation time had no large effect on the variance of the physiological rate of freshwater organisms (Noble et al. 2025), the variability in the detected effects tends to suggest that the effect of acclimation on the variance of the physiological rate was highly study‐dependent. Our study tends to confirm this pattern. Therefore, despite we found mean scaling being very different among conditions (0.591 up to 1.115), the high confidence intervals prevented detecting effects of our treatment. For instance, the scaling exponent for two conditions (cold‐cold and warm‐warm) was high and close to the value described at the intraspecific level in fish species (i.e., 0.89, Jerde et al. 2019). Yet, the high confidence intervals (Figure 1b), also found in other studies (see Jerde et al. 2019 for a review), encompassed the 3/4 allometric exponent classically described (Gillooly 2001; Glazier 2005). Further studies may aim at teasing apart the role of growth and activity on energy costs for maintenance in relation to temperature.

Studying the thermal physiology of stenothermic species is essential to assess their potential evolution in warmer and less predictable environments. Our findings indicate that the metabolic plasticity of Alpine charr will result in a higher metabolic rate, regardless of the acclimation time to warm temperatures in one of the southernmost populations (Lake Geneva). Young stages in Alpine charr are known to be sensitive to temperature, with studies showing that temperature increases and abrupt variations can increase mortality or lead to changes in life‐history traits and morphology, particularly in the population of Lake Geneva (e.g., Lubin et al. 2024; Mari et al. 2021). We here found that Q10 we relatively high for both acclimation conditions, confirming that Arctic charr displayed high thermal sensitivity as found in other studies (Huuskonen et al. 2003). Moreover, the temperature of Lake Geneva is expected to increase dramatically in the next decades (Desgué‐Itier et al. 2023). Since metabolism is strongly dependent upon temperature and is a key trait linked to multiple ecological processes, warming will likely result in higher metabolic rates with consequences for reproduction capacities and ecological interactions (Brown et al. 2004). Therefore, additional studies will be needed to evaluate the impact of the thermal plasticity of metabolic rate on the population dynamics of Alpine charr.

To conclude, our findings suggest a strong metabolic plasticity to temperature, as shown by the significant effect of increasing temperature, regardless of the time of exposure. Acclimation time to a thermal environment is classically expected to buffer the impact of temperature on a physiological rate, a pattern that we did not confirm in this study. The relationship between metabolic rate and body mass also seemed to be conserved across temperature treatments, although high variability in the data prevented strong conclusions. While some internal factors (such as activity) may explain this lack of response, it may also suggest that cellular and mechanical constraints on metabolism are strong in Alpine charr. While temperature is dramatically increasing in lakes, future studies may focus on the consequences of the thermal dependency of metabolism on ecological variables (e.g., reproduction capacities) to better understand the future evolution of Alpine charr.

## Author Contributions

**Allan Raffard:** conceptualization (lead), formal analysis (lead), funding acquisition (equal), writing – original draft (lead). **Martin Daufresne:** conceptualization (supporting), funding acquisition (equal), methodology (supporting), writing – review and editing (supporting). **Jean Guillard:** funding acquisition (equal), writing – review and editing (equal). **François‐Raphaël Lubin:** conceptualization (supporting), investigation (lead), methodology (lead), writing – review and editing (equal). **Emilie Réalis‐Doyelle:** methodology (supporting), writing – review and editing (equal). **Hervé Rogissart:** conceptualization (supporting), writing – review and editing (equal). **Loïc Teulier:** conceptualization (supporting), methodology (supporting), writing – review and editing (equal).

## Conflicts of Interest

The authors declare no conflicts of interest.

## Supporting information


**Data S1:** ece371907‐sup‐0001‐Supinfo.docx.

## Data Availability

Data are available on Figshare: https://doi.org/10.6084/m9.figshare.24968040.v1.
