# Supplementary material for: Effect of Time of Temperature Exposure on Routine Metabolic Rate and Body Mass Scaling in Alpine Charr (Salvelinus umbla)
Source: Ecol Evol. 2025 Aug 6;15(8):e71907. doi: 10.1002/ece3.71907 (PMC12328023; doi:10.1002/ece3.71907)
Supplement: Supplementary file 1 — Data S1: ece371907‐sup‐0001‐Supinfo.docx. [file ECE3-15-e71907-s001.docx]

**Supplementary information**

Effect of time of temperature exposure on routine metabolic rate and body mass scaling in Alpine charr (*Salvelinus umbla*).

Allan Raffard^1,2^, Martin Daufresne^2,3^, Jean Guillard^1,2^, François-Raphaël Lubin^1,2^, Emilie Réalis-Doyelle^1^, Hervé Rogissart^1^, Loïc Teulier^4^

*^1^Univ. Savoie Mont Blanc, INRAE, CARRTEL, 74200 Thonon-les-Bains, France*

*^2^Pôle ECLA (OFB, INRAE, USMB), 74200 Thonon-les-Bains, France^,^*

*^3^INRAE, Aix-Marseille Univ., RECOVER, Aix-en-Provence, France*

*^4^Universite Claude Bernard Lyon 1, CNRS, ENTPE, LEHNA UMR 5023, Villeurbanne, F-69100, France*

**Author for correspondence:** Allan Raffard

E-mail: [allan.raffard@inrae.fr](mailto:allan.raffard@inrae.fr)

**Table S1.** Body size (total length) of reproductive individuals Alpine charr (*Salvelinus umbla*) used to generate the five full-sib families.

| Family | Sex | Size (mm) |
| --- | --- | --- |
| L1 | Male | 320 |
|  | Female | 390 |
| L3 | Male | 320 |
|  | Female | 385 |
| L4 | Male | 390 |
|  | Female | 340 |
| L8 | Male | 380 |
|  | Female | 440 |
| L10 | Male | 430 |
|  | Female | 460 |

**Table S2.** Number of eggs of Alpine charr (*Salvelinus umbla*) from Lake Geneva incubated at each temperature with mortality and hatching rates

| Incubation temperature | Family | Total  eggs | Dead  eggs | Hatch |
| --- | --- | --- | --- | --- |
| 4.5°C | L1 | 97 | 5 | 92 |
| 4.5°C | L3 | 69 | 14 | 55 |
| 4.5°C | L4 | 90 | 18 | 72 |
| 4.5°C | L8 | 77 | 11 | 66 |
| 4.5°C | L10 | 80 | 7 | 73 |
| 8.5°C | L1 | 92 | 10 | 82 |
| 8.5°C | L3 | 90 | 21 | 69 |
| 8.5°C | L4 | 82 | 13 | 69 |
| 8.5°C | L8 | 81 | 28 | 53 |
| 8.5°C | L10 | 104 | 38 | 66 |

**
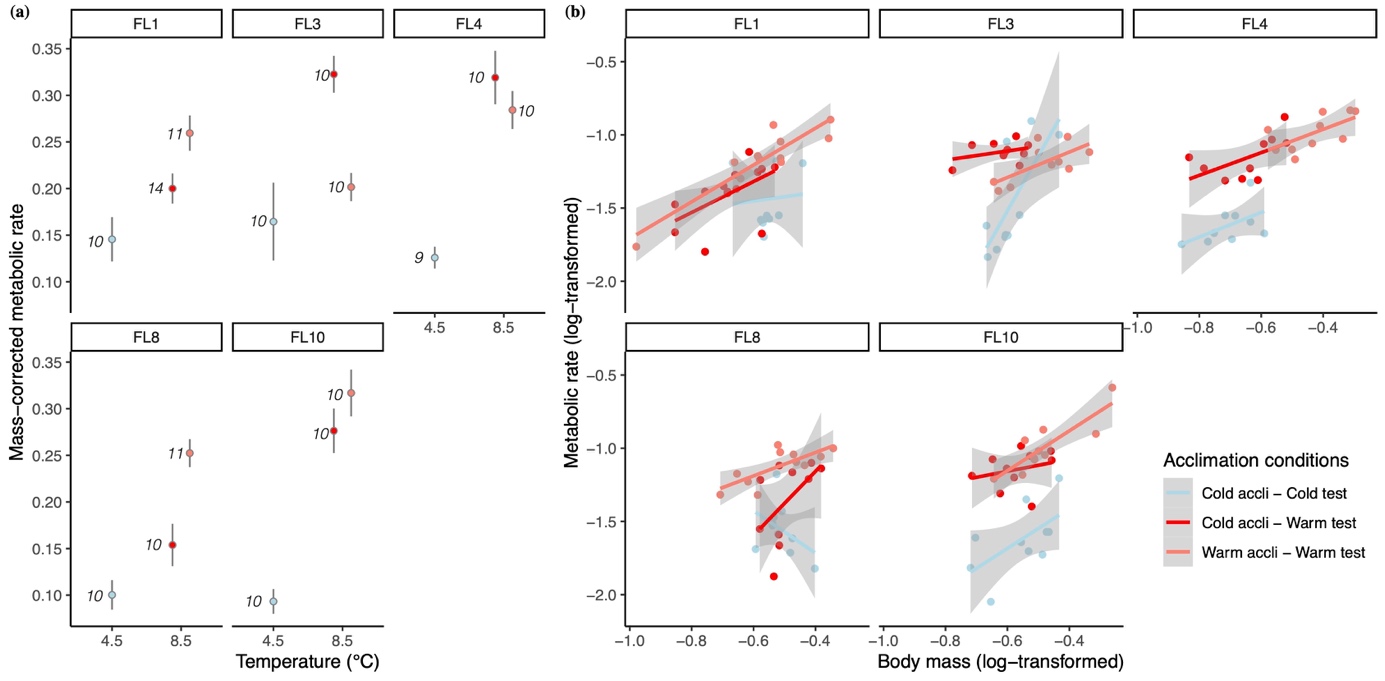
**

**Figure S1. (a)** Effect of temperature and acclimation temperature on metabolic rate (mgO_2_.h^-1^.g^-1^) of Alpine charr (*Salvelinus umbla*) in each family. Error bars represent ± 1 standard error SE. The numbers next to the points represent the numbers of individuals tested in each treatment for each family. **(b)** Relationship between body mass (g, log-transformed) and metabolic rate (mgO_2_.h^-1^, log-transformed) in each of the acclimation conditions for each tested family. Shading areas represent 95% confident intervals CIs. Blue points, error bars and slopes represent the cold acclimation - cold test condition; dark red points, error bars and slopes represent cold acclimation – warm test condition; and the light red points, error bars and slopes represent the warm acclimation – warm test conditions. The regression equations were calculated based on linear model (numbers into brackets are SE).
